# Supplementary material for: Epidemiology of Simultaneous Medullary and Papillary Thyroid Carcinomas (MTC/PTC): An Italian Multicenter Study
Source: Cancers (Basel). 2019 Oct 9;11(10):1516. doi: 10.3390/cancers11101516 (PMC6826384; doi:10.3390/cancers11101516)
Supplement: Supplementary file 1 [file cancers-11-01516-s001.pdf]

Supplementary Materials: Epidemiology of Simultaneous Medullary and Papillary Thyroid Carcinomas (MTC/PTC): An Italian, Multicenter Study

Marialuisa Appetecchia, Rosa Lauretta, Agnese Barnabei, Letizia Pieruzzi, Irene Terrenato, Elisabetta Cavedon, Caterina Mian, Maria Grazia Castagna, Rossella Elisei; on behalf of the SIE (Italian Society of Endocrinology) Working Group

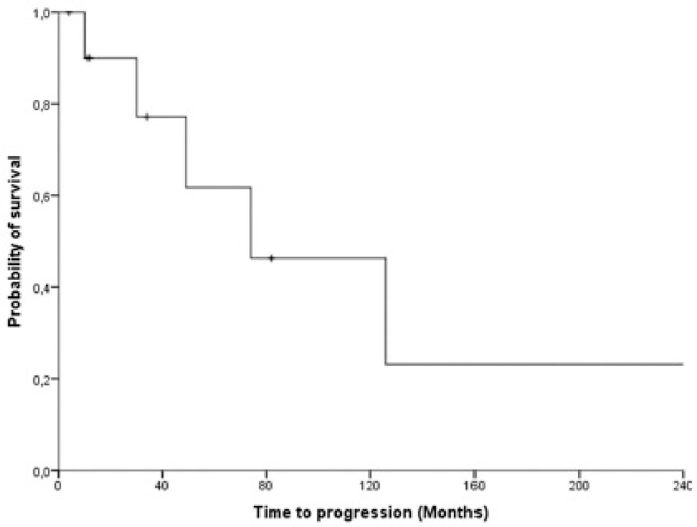

Median time to progression 74 months, 95%CI 0-148

(**A**)

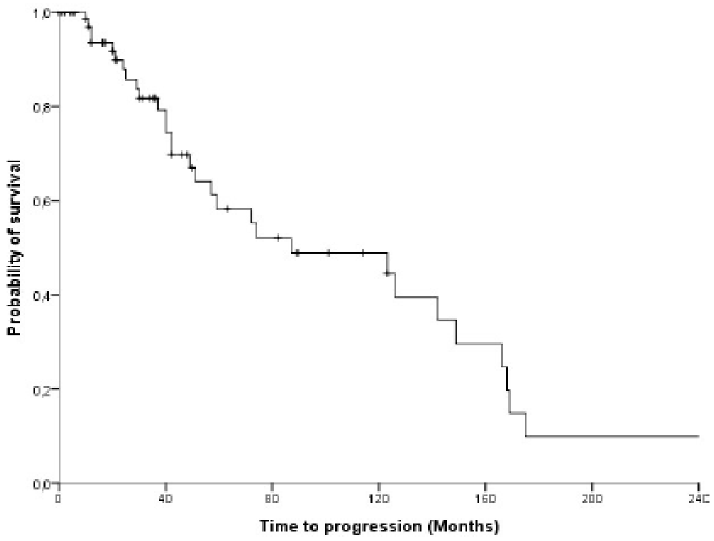

Median time to progression 87 months, 95%CI 11.5-162.5

(**B**)

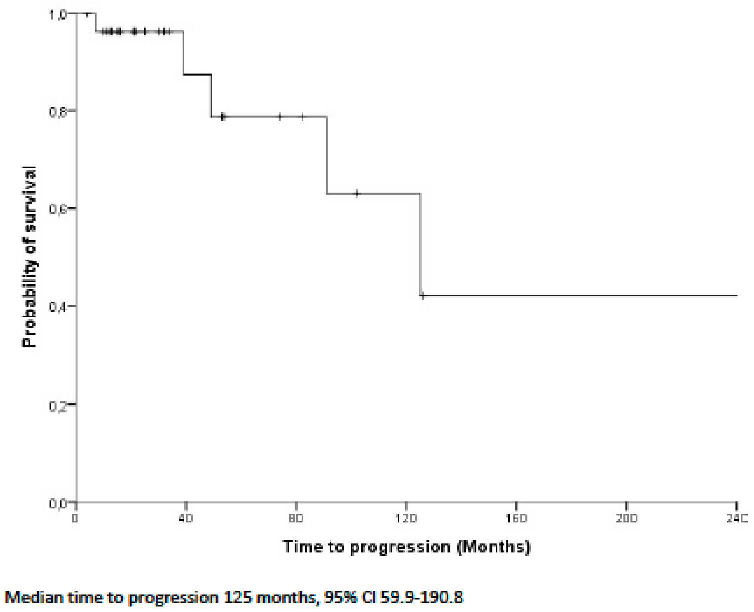

(**C**)

Figure S1. Analysis of survival by tumor size. Panel (**A**) Both PTC and MTC >1 cm (*N* = 11). Panel (**B**) MTC >1 cm (*N* = 78). Panel (**C**) PTC>1 cm (*N* = 27).

Table S1. Cancer site of the 21 patients who had concomitant cancer comorbidity.

Patient	1st Cancer	2nd Cancer
1	Prostate	Colon
2	Breast	
3	Breast	
4	Breast	
5	Breast	
6	Breast	Melanoma
7	Kidney	Bowel
8	Melanoma	
9	Pheochromocytoma	
10	Pheochromocytoma	
11	Pheochromocytoma	
12	Prostate	
13	Lymphoma	
14	Leukemia (pediatric age)	
15	Melanoma	
16	Melanoma	
17	Mesothelioma	Pancreas
18	Multiple myeloma	
19	Breast	Neuroblastoma
20	Kidney	
21	Sarcoma Ewing	

Table S2. RET mutations.

RET	*N*	%	Germinal *
**RET (–)**	88	48.1	
**RET (+)**	24	13,1	
unknown	6	*25,0*	
A883F	1	*4,2*	*1*
C620S	2	*8,3*	*1*
C630T	1	*4,2*	

del631E	1	*4,2*	
E632_I638del	1	*4,2*	
M918T	2	*8,3*	*1*
D898_E901del	1	*4,2*	
S891A	1	*4,2*	
V804M	8	*33,3*	*2*
**RET unknown**	71	38,8	

* The mutation was germinal in 5/24 subjects (21%) and somatic in the remaining 19 subjects.

© 2019 by the authors. Licensee MDPI, Basel, Switzerland. This article is an open access article distributed under the terms and conditions of the Creative Commons Attribution (CC BY) license (http://creativecommons.org/licenses/by/4.0/).
